# Additional Value of Cardiac Assessment With Oncologic ^18^F-FDG PET/CT in Pre-Treatment Tumor Patients

**DOI:** 10.31083/RCM46885

**Published:** 2026-05-25

**Authors:** Runlong Lin, Yanwen Bing, Jin Wang, Aijuan Tian, Jing Yu, Wenli Xie

**Affiliations:** ^1^Department of Nuclear Medicine, The Second Hospital of Dalian Medical University, 116023 Dalian, Liaoning, China; ^2^Department of Health Management Center, The Second Hospital of Dalian Medical University, 116023 Dalian, Liaoning, China; ^3^Department of Vascular Surgery, The Second Hospital of Dalian Medical University, 116023 Dalian, Liaoning, China; ^4^Department of Cardiovascular Medicine, The Second Hospital of Dalian Medical University, 116023 Dalian, Liaoning, China

**Keywords:** cardio-oncology, ^18^F-FDG PET/CT, cardiac assessment, abnormal uptake

## Abstract

**Background:**

To study the occurrence, site, reasons, metabolism, and texture features of unusual heart absorption in cancer patients undergoing their first Fluorine-18 Fluorodeoxyglucose Positron Emission Tomography/Computed Tomography (^18^F-FDG PET/CT) full-body scan.

**Methods:**

A total of 2159 patients who underwent their first ^18^F-FDG PET/CT full-body scan and received hospital care between July 2017 and December 2022 were included. Of these, 1611 were diagnosed with tumors, and 548 were non-tumor cases, based on clinical, pathological, and PET/CT evaluations. Abnormal uptake was categorized as a “known cause” when supported by clinical evidence of the associated origin and as an “unknown cause” when evidence was insufficient. We compared metabolic parameters and texture features between groups and constructed receiver operating characteristic (ROC) curves.

**Results:**

Among the patients studied, 118 with tumors exhibited abnormal cardiac uptake patterns, while 14 non-tumor patients showed similar abnormalities. The occurrence of abnormal cardiac uptake without a known cause among cases of abnormal cardiac uptake was higher in tumor patients than in non-tumor patients (62.71% vs. 35.71%; *p* > 0.05). Within the tumor group, the high-tumor-burden subset had a significantly higher proportion of unexplained abnormal cardiac uptake than the low-tumor-burden subset (72.9% vs. 52.5%; *p* < 0.05). ROC curve analysis revealed that a combined assessment of selected metabolic parameters and texture features yielded an area under the curve (AUC) of 0.809 (0.732, 0.886), sensitivity of 77.3%, and specificity of 71.6%.

**Conclusion:**

Unusual cardiac uptake observed in tumor patients during ^18^F-FDG PET/CT scans could be linked to existing heart conditions and the extent of the tumor. Metabolic parameters and texture characteristics provide valuable insights into the underlying causes.

## 1. Introduction

Cancer and cardiovascular diseases stand as leading causes of mortality. Advances in medical technology have notably reduced cancer-related deaths through chemotherapy, enhancing patient outcomes—a pivotal aspect of effective tumor care [1]. Nevertheless, chemotherapy drugs can harm various organs, with cardiotoxicity being a notable concern [2]. Detecting this early is crucial for both oncologists and cardiologists.

Given that chemotherapy’s cardiotoxic effects span the myocardium, blood vessels, cardiac rhythm, and blood pressure, evolving cardiovascular imaging methods aid in spotting and managing such cardiotoxicity. Techniques like Single-Photon Emission Computed Tomography (SPECT) and positron emission tomography (PET) offer significant value in spotting abnormal heart perfusion and evaluating ischemic risks [3]. PET molecular imaging, pivotal in oncology, not only aids in diagnosis but also directs treatment and monitors its efficacy. The widely used tracer in PET/CT applications, Fluorine-18 Fluorodeoxyglucose (^18^F-FDG), distinguishes tumor tissues based on distinct metabolic activity from surrounding areas. In cardiovascular contexts, it gauges inflammation and ischemia by assessing heightened metabolic activity and glucose uptake in the heart and vessels [4]. While preparation methods differ between tumor and heart imaging, a prolonged fast before tumor scans minimizes skeletal and heart muscle glucose uptake, leaving ischemia and inflammation-related uptake unaffected [5, 6]. Despite guidelines emphasizing the role of cardiac nuclear imaging in recognizing and managing tumor-related heart issues during treatment [7], not all cancer patients undergo standardized cardiac nuclear tests. Hence, analyzing abnormal cardiac uptake in Fluorine-18 Fluorodeoxyglucose Positron Emission Tomography/Computed Tomography (^18^F-FDG PET/CT) scans of tumor patients remains a significant area for exploration.

In ^18^F-FDG PET/CT scans of tumor patients, we noted varying patterns and intensities of abnormal cardiac ^18^F-FDG uptake both pre- and post-radiotherapy and chemotherapy. Some cases of abnormal uptake align with existing heart conditions, while others lack a clear cause. Understanding these uptake patterns is crucial to avoid misdiagnoses and lay the groundwork for effectively using ^18^F-FDG PET/CT to pinpoint cardiotoxicity related to tumors or cancer treatments. Currently, research on abnormal cardiac uptake prior to anticancer interventions in standard oncologic ^18^F-FDG PET/CT scans is sparse. Hence, leveraging imaging and clinical data, this study seeks to delineate the features and clinical relevance of abnormal cardiac uptake preceding anticancer treatments in oncologic ^18^F-FDG PET/CT evaluations.

## 2. Materials and Methods

### 2.1 Study Subjects

From July 2017 to December 2022, we analyzed 2159 cases at the Second Hospital of Dalian Medical University. These patients underwent their initial ^18^F-FDG PET/CT scans for reasons like unexplained elevated tumor markers, tumor diagnosis, preoperative staging, or unexplained fever before receiving treatment at our hospital. Based on clinical, pathological, and PET/CT findings, we categorized them into two groups: 1611 with newly diagnosed tumors and 548 without tumors. A non-tumor patient refers to an individual in whom no malignant tumor lesions are found through clinical diagnosis, including methods such as medical history collection, physical examination, laboratory tests, imaging examinations, and pathological diagnosis. Exclusion criteria included: (1) Poor quality of PET/CT images; (2) Incomplete collection of patient case data; (3) History of previous tumors and receipt of radiation or chemotherapy; (4) The fasting blood glucose level exceeded 11.1 mmol/L on the day of examination; (5) Severe liver or kidney dysfunction (Child-Pugh Class C‌ or CKD phases 4–5); (6) Patients with heart-related lesions (such as tumor or sarcoidosis, etc.). Comprehensive data, including medical history, echocardiography, ECG, cardiac biomarkers, and brain natriuretic peptide tests, were accessible. This research received approval from the Ethics Committee of the Second Hospital of Dalian Medical University (No.2019-049), and all experiments were performed in accordance with relevant guidelines and regulations. All individual participants included in the study provided signed informed consent.

### 2.2 Collection of Clinical Data

We gathered comprehensive patient clinical data, encompassing age, gender, hypertension, diabetes, and heart disease history, along with the purpose of PET/CT scans, blood test results, and ultimate disease diagnoses. Additionally, we compiled cardiac imaging information, including electrocardiograms, echocardiography findings, coronary CT scans, and coronary angiography outcomes.

### 2.3 ^18^F-FDG PET/CT Examination

We utilized the Philips Ingenuity TF PET/CT scanner (Cleveland, OH, USA) for the evaluations. The ^18^F-FDG was generated and synthesized using the Sumitomo HM-10 cyclotron accelerator and the chemical synthesis module from PET Co., Ltd. (Beijing), ensuring a radiochemical purity exceeding 95%. Patients refrained from eating for a minimum of 12 hours before the procedure. Following the administration of ^18^F-FDG at a dosage of 3.7–5.55 MBq/kg, patients rested in a dimly lit room for 60 minutes before undergoing PET/CT scans post bladder voiding. The scan encompassed from the skull base to the upper-mid thigh. Initially, CT scans were conducted under the parameters: voltage set at 120 kV, current at 90 mA, rotation speed of 0.75 s/rotation, and a matrix of 512 × 512. Subsequently, PET imaging followed with conditions set at a matrix of 144 × 144 and 1-minute acquisition for each bed position, totaling 8–10 bed positions. Post attenuation correction and OSEM reconstruction, PET images were aligned with CT images on the image processing workstation.

### 2.4 Image Analysis

#### 2.4.1 Myocardial Glucose Uptake Analysis

Following the guidelines from the American Society of Nuclear Cardiology [8] and integrating insights from prior research [9, 10], two seasoned nuclear medicine physicians retrospectively analyzed the myocardial glucose uptake images from ^18^F-FDG PET/CT scans. Through visual examination, they categorized myocardial glucose uptake patterns into four distinct types:

(1) No uptake: This category indicates that overall uptake in the left ventricular myocardium is either equivalent to or less than that in the blood pool.

(2) Diffuse uptake: Here, the left ventricular myocardium displays a generally uniform uptake pattern without notable focal or elevated uptakes.

(3) Focal uptake: This type is characterized by specific areas within the left ventricular myocardium exhibiting high uptake, while the rest remains at levels comparable to or lower than the blood pool.

(4) Focal uptake on diffuse uptake background: In this scenario, certain segments of the myocardium exhibit heightened uptake against a backdrop of diffuse uptake throughout the left ventricular myocardium. For a visual reference, please see Fig. 1.

**Fig. 1.**
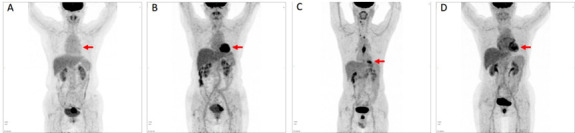
**Patterns of myocardial glucose uptake (red arrow)**. (A) No FDG Uptake. (B) Diffuse FDG Uptake. (C) Focal FDG Uptake. (D) Focal FDG Uptake on Diffuse Background.

Based on the myocardial glucose uptake patterns, locations, and characteristics, we can categorize them into either normal or abnormal myocardial uptake [10]. Abnormal myocardial uptake is characterized by:

(1) Focal uptake outside the basal and papillary muscle regions of the left ventricle.

(2) Uneven uptake in the left ventricle’s lateral wall.

(3) Higher uptake in the right ventricle compared to the left ventricle.

(4) Greater atrial uptake than blood pool uptake when the left ventricle shows minimal or no uptake.

(5) Excluding standard uptake in specific areas like fatty hypertrophy of the atrial septum, right atrial septal ridge, among others.

#### 2.4.2 Determination of the Causes of Abnormal Myocardial Uptake

Using patients’ overall clinical data, medical histories, and cardiac imaging findings, we investigated potential reasons for abnormal myocardial uptake. If abnormal cardiac uptake can be correlated with the patient’s previous history of heart disease and other abnormal findings in cardiac imaging, we classify it as a “known cause” of abnormal uptake. Conversely, if clinical data didn’t provide adequate support for pinpointing the cause, we labeled it as an “unknown cause” of abnormal uptake.

#### 2.4.3 Quantitative Analysis of Abnormal Myocardial Uptake

We utilized the MEMRS‑NM‑4.0.008 (Beijing MedEx Technology Co., Ltd., Beijing, China) to determine the maximal SUV in the heart (SUVmax-heart), average SUV of the heart (SUVmean-heart), and the heart-to-blood pool SUV ratio (heart/ascending aorta) within areas displaying abnormal myocardial uptake on integrated PET/CT images. Additionally, measurements included the whole-body tumor metabolic volume (MTV) and total lesion glycolysis (TLG). Employing the relative threshold method set at 41% of the tumor lesion’s SUVmax [11], the software autonomously identified regions of interest (ROI) along the lesion periphery, subsequently computing both MTV and TLG for the tumor. Based on the median TLG value, patients with tumors were segmented into high and low TLG groups.

#### 2.4.4 Texture Analysis of Abnormal Myocardial Uptake PET/CT Images

We employed MaZda 4.6 (The Technical University of Lodz, Lodz, Poland, available at https://qmazda.p.lodz.pl/index.php?action=mazda_46) for texture analysis. Initially, color PET/CT fused images underwent conversion to grayscale to facilitate texture analysis [12, 13, 14, 15]. The primary slice featuring the abnormal uptake lesion was saved in a bitmap image file (BMP) format and then imported into MaZda. Before feature extraction, the µ ± 3σ method (where µ is the mean of image gray values and σ is the standard deviation of image gray values) is adopted to perform unified normalization preprocessing on the image, so as to reduce the impact of brightness and contrast on the image gray values. Here, two physicians independently outlined ROI and identified texture features. On PET/CT images, the ROI is gradually delineated along the edge of the area with abnormal FDG uptake in the heart. MaZda offers six texture feature categories in 2D mode analysis: grey-level run-length matrix (GLRM), absolute gradient (GRA), histogram, gray-level co-occurrence matrix (GLCM), wavelet transform (WAV), and auto-regressive model (ARM), encompassing a total of 275 texture features. We utilized the intra-class correlation coefficient (ICC) to assess the consistency between physicians; features with an ICC exceeding 0.75 progressed to subsequent statistical analyses. For dimensionality reduction, we employed algorithms such as Fisher (F), POE+ACC (a blend of classification error probability and average correlation coefficients), and mutual information (MI). Each algorithm autonomously identified the ten most distinguishing texture features, culminating in a potential selection of 30 texture features when combined.

### 2.5 Statistical Analysis 

We utilized the MaZda software B11 program for discriminant analysis of selected texture parameters, employing methods like raw data analysis (RDA), principal component analysis (PCA), linear discriminant analysis (LDA), and nonlinear discriminant analysis (NDA). Results from these analyses were quantified as misclassification rates (R), representing the proportion of incorrectly classified lesions to the total.

For statistical evaluations, we employed SPSS Statistics 26.0 and R3.4.3 software. The normality of continuous variables was assessed using the Kolmogorov-Smirnov test. Normally distributed data are presented as mean ± standard deviation ($\bar{X}$ ± S), while non-normally distributed data appear as median (P25, P75). Categorical variables are depicted as frequencies and percentages (%). The ICC evaluated the agreement between physicians on texture parameter measurements. To discern differences between two independent samples, we used the *t*-test or the Mann-Whitney U test. Meanwhile, the chi-square test compared rates among these samples. Multivariate logistic regression analysis (stepwise method) was used to establish a model for variable selection and intergroup comparison predictive factors to distinguish between groups. Lastly, indicators with a *p*-value less than 0.05, SUVmax-heart, SUVmean-heart and the heart-to-blood pool SUV ratio were included in the analyses and plotted on a receiver operating characteristic (ROC) graph, yielding metrics like sensitivity, specificity, cutoff value, and area under the curve (AUC) for each feature measured by the C-statistic used to quantify predictive power. The optimal cut-off point is commonly determined using the “Youden index”, which is calculated as sensitivity + specificity–1. We conducted a bootstrapping internal validation with 1000 bootstrap resamples to check for overfitting and calculated a relatively corrected C-index. A significance threshold was set at *p* < 0.05.

## 3. Results

### 3.1 Clinical Data of 2159 Patients Undergoing ^18^F-FDG PET/CT Imaging 

This study encompassed 2159 patients who underwent ^18^F-FDG PET/CT imaging. Of these, 1611 patients received a tumor diagnosis based on clinical and pathological evaluations, while 548 were non-tumor cases. The tumor-afflicted patients exhibited notably higher rates of age, diabetes, coronary heart disease, and hypertension compared to their non-tumor counterparts, with statistically significant variances (*p* < 0.05). Conversely, gender, atrial fibrillation, heart valve disease, and prior history of heart failure showed no significant differences between the two groups (*p* > 0.05) (Table 1).

**Table 1.** **Clinical data of 2159 patients undergoing ^18^F-FDG PET/CT imaging**.

		Tumor patients (N = 1611)	Non-tumor patients (N = 548)	*p*-value
Age (years)		61.60 ± 12.65	58.46 ± 14.22	0.001
Male (n, %)		870 (54.00)	280 (51.10)	0.238
Diabetes (n, %)		201 (12.48)	46 (8.39)	0.009
Pre-existing cardiovascular diseases (n, %)				
	Coronary artery disease	98 (6.08)	16 (2.92)	0.004
	Atrial fibrillation	39 (2.42)	9 (1.64)	0.286
	Valvular heart disease	4 (0.25)	2 (0.36)	0.654
	Heart failure	8 (0.50)	7 (1.28)	0.057
	Hypertension	456 (28.31)	66 (12.04)	0.001

^18^F-FDG PET/CT, Fluorine-18 Fluorodeoxyglucose Positron Emission Tomography/Computed Tomography.

### 3.2 Cardiac Uptake Analysis of 2159 Patients Undergoing ^18^F-FDG PET/CT Imaging

In the analysis of myocardial glucose uptake patterns among 2159 patients who underwent ^18^F-FDG PET/CT imaging, tumor patients exhibited markedly reduced diffuse uptake in the heart compared to those without tumors. Conversely, tumor patients displayed notably elevated focal uptake in the heart relative to non-tumor patients, indicating statistically significant distinctions (*p* < 0.05). However, the occurrence of patients displaying no uptake and those manifesting a focal-on-diffuse uptake pattern showed no noteworthy variances between the groups (*p* > 0.05). Furthermore, considering the patterns, locations, and characteristics of myocardial glucose uptake, abnormalities were observed in 118 tumor patients versus 14 non-tumor patients, highlighting significant disparities between these cohorts (*p* < 0.05) (Table 2).

**Table 2.** **Cardiac uptake analysis of 2159 patients undergoing ^18^F-FDG PET/CT imaging**.

		Tumor patients (N = 1611)	Non-tumor patients (N = 548)	*p*-value
Left ventricle uptake pattern (n, %)				
	No uptake	870 (54.00)	273 (49.82)	0.09
	Diffuse uptake	327 (20.30)	231 (42.15)	0.001
	Focal uptake	353 (21.91)	29 (5.29)	0.001
	Focal on diffuse uptake	61 (3.79)	15 (2.74)	0.25
Cardiac abnormal uptake (n, %)		118 (7.32)	14 (2.55)	0.001

### 3.3 Analysis of the Location and Causes of Abnormal Cardiac Uptake in Patients Undergoing ^18^F-FDG PET/CT Imaging

In the evaluation of 132 patients manifesting abnormal cardiac uptake on ^18^F-FDG PET/CT scans, 118 were tumor patients, and 14 were non-tumor patients. Within the tumor cohort, 85 cases (72.03%) exhibited abnormal uptake in the left ventricle, 3 cases (2.54%) in the right ventricle, and 30 cases (25.42%) in the atrium. Conversely, among non-tumor patients, 7 cases (50%) displayed abnormal uptake in the left ventricle, 3 cases (21.43%) in the right ventricle, and 4 cases (28.57%) in the atrium.

Upon analyzing patients’ clinical data, medical histories, and cardiac imaging findings, potential causes for the abnormal cardiac uptake were deliberated. Abnormal uptake in the left ventricle might arise from conditions like myocardial ischemia or hypertrophic cardiomyopathy. Similarly, abnormal uptake in the right ventricle could be attributed to pulmonary arterial hypertension or valvular diseases, while atrial uptake abnormalities might stem from conditions such as atrial fibrillation, atrial enlargement due to hypertension, or pulmonary arterial hypertension.

Notably, among tumor patients, 74 cases (62.71%) presented with an unknown cause, whereas in the non-tumor group, 5 cases (35.71%) had an unidentified cause. This discrepancy indicates a higher rate of unknown causes for abnormal uptake in tumor patients compared to their non-tumor counterparts, although with no statistical significance (Table 3). Fig. 2 illustrates examples of abnormal cardiac uptake in patients as observed in ^18^F-FDG PET/CT imaging.

**Table 3.** **Analysis of the location and causes of abnormal cardiac uptake in 132 patients undergoing ^18^F-FDG PET/CT imaging**.

Location and Cause (n, %)		Tumor patients (N = 118)	Non-tumor patients (N = 14)	*p*-value
Left ventricle		85 (72.03)	7 (50)	0.09
	Myocardial ischemia	7 (5.93)	4 (28.57)	0.004
	Hypertensive ventricular hypertrophy	14 (11.86)	1 (7.14)	0.599
	Heart failure	-	1 (7.14)	-
	Unknown cause	64 (54.24)	1 (7.14)	0.001
Right ventricle		3 (2.54)	3 (21.43)	0.001
	Pulmonary arterial hypertension	2 (1.69)	1 (7.14)	0.196
	Valvular disease	-	1 (7.14)	-
	Unknown cause	1 (0.85)	1 (7.14)	0.068
Atrium		30 (25.42)	4 (28.57)	0.799
	Atrial fibrillation	13 (11.02)	1 (7.14)	0.656
	Hypertensive atrial enlargement	6 (5.08)	-	-
	Pulmonary arterial hypertension	2 (1.69)	-	-
	Unknown cause	9 (7.63)	3 (21.43)	0.089
Total unknown cause		74 (62.71)	5 (35.71)	0.051

**Fig. 2.**
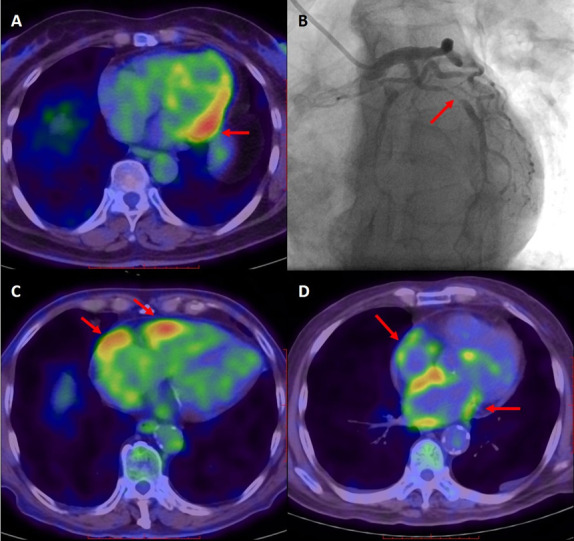
**Illustrative cases of abnormal cardiac uptake in tumor patients via ^18^F-FDG PET/CT imaging**. (A,B) A 60-year-old female presented with angina symptoms, and coronary angiography revealed a 99% stenosis in the mid-segment of the left circumflex artery (indicated by arrow). Concurrently, elevated tumor markers were observed. The ^18^F-FDG PET/CT scan displayed heightened focal uptake in the left ventricular lateral wall (arrow), suggesting myocardial ischemia as the cause for the abnormal uptake. (C) A 79-year-old male’s ^18^F-FDG PET/CT scan showcased increased uptake in both the right ventricular and right atrial walls (arrow). Subsequent cardiac ultrasound indicated rheumatic heart disease, severe mitral valve stenosis with significant regurgitation, mild aortic valve regurgitation, bi-atrial enlargement, moderate tricuspid valve regurgitation, and moderate pulmonary artery hypertension. These findings collectively suggest that the abnormal uptake in the right ventricular and right atrial walls stems from valvular issues and pulmonary artery hypertension. (D) An 80-year-old male’s ^18^F-FDG PET/CT scan revealed amplified diffuse uptake in both atrial walls (arrow). The electrocardiogram confirmed atrial fibrillation and right bundle branch block. Consequently, the abnormal uptake observed in both atrial walls is attributed to atrial fibrillation.

### 3.4 Comparison of Cardiac Uptake With Unknown Cause in Tumor Patients Undergoing ^18^F-FDG PET/CT Imaging in Different TLG Groups

In a cohort of 118 tumor patients who underwent ^18^F-FDG PET/CT imaging and TLG assessment, they were categorized into high and low TLG groups based on the median value. Within the high TLG group, 43 patients (72.9%) exhibited cardiac uptake of unknown origin. In contrast, the low TLG group had 31 patients (52.5%) showcasing cardiac uptake with an undetermined cause. Significantly, the incidence of cardiac uptake of unknown cause was notably elevated in the high TLG group compared to the low TLG group, with statistical significance observed (*p* < 0.05) (Table 4).

**Table 4.** **Comparative analysis of cardiac uptake with ambiguous etiology among tumor patients subjected to PET/CT imaging across different TLG categories**.

	High TLG group (N = 59)	Low TLG group (N = 59)	*p*-value
Cardiac abnormal uptake with unknown cause (n, %)	43 (72.9%)	31 (52.5%)	0.022

TLG, total lesion glycolysis.

### 3.5 Analysis of Metabolic Parameters of Abnormal Cardiac Uptake in Tumor Patients Using ^18^F-FDG PET/CT

An analysis was undertaken to compare the metabolic parameters of abnormal cardiac uptake lesions among 118 tumor patients using ^18^F-FDG PET/CT. The results indicated that there were no statistically significant variances in SUVmax-heart, SUVmean-heart, and heart-to-blood pool SUV ratio between the subset with unknown causes for abnormal uptake and those with identifiable causes (*p* > 0.05). For an in-depth examination of the metabolic parameters related to abnormal cardiac uptake in tumor patients who underwent PET/CT imaging, please refer to Table 5.

**Table 5.** **Comprehensive evaluation of metabolic parameters associated with abnormal cardiac uptake in 118 tumor patients subjected to PET/CT imaging**.

Metabolic parameter	Unknown cause of abnormal uptake (N = 74)	Known cause of abnormal uptake (N = 44)	*p*-value
SUVmax-heart	4.43 ± 1.29	4.69 ± 1.53	0.337
SUVmean-heart	2.54 ± 0.66	2.62 ± 0.80	0.576
Heart-to-blood pool SUV ratio	2.28 ± 0.84	2.15 ± 0.62	0.403

### 3.6 Analysis of Discrimination Results Based on Texture Features

Inter-observer consistency for the two physicians analyzing texture features of PET/CT images was evaluated using the ICC, which ranged between 0.75 and 0.99. These values signify excellent agreement between the observers across all texture features assessed. Additionally, by integrating four discrimination analysis techniques available in the B11 program with three distinct dimensionality reduction algorithms, the MI+PA+F dimensionality reduction approach combined with the NDA discrimination method yielded the most favorable outcome. Specifically, this combination achieved the lowest misclassification rate at 12.71% (Table 6).

**Table 6.** **Comparative analysis of misclassification rates for differentiating between known and unknown causes of abnormal uptake utilizing various dimensionality reduction algorithms and discrimination techniques**.

Dimensionality reduction method	RDA	PCA	LDA	NDA
Fisher (F)	44.07%	46.61%	33.05%	23.73%
POE+ACC (PA)	45.76%	51.69%	41.53%	21.19%
MI	36.44%	37.29%	35.59%	16.10%
MI+PA+F	50%	47.46%	32.20%	12.71%

RDA, raw data analysis; PCA, principal component analysis; LDA, linear discriminant analysis; NDA, nonlinear discriminant analysis.

### 3.7 Texture Analysis of Abnormal Cardiac Uptake in Tumor Patients Using ^18^F-FDG PET/CT Imaging

Thirty texture features were obtained using the MI+PA+F method following dimensionality reduction. Among these features, θ3 (Theta 3), Horizontal Run Length Nonuniformity (Horzl_RLNonUni), θ2 (Theta 2), S(1,0) Entropy, Run Length Nonuniformity at 135 degrees (135dr_RLNonUni), Run Length Nonuniformity at 45 degrees (45dgr_RLNonUni), Horizontal Grey-Level Nonuniformity (Horzl_GLevNonU), Grey-Level Nonuniformity at 45 degrees (45dgr_GLevNonU), Run Length Nonuniformity in the vertical direction (Vertl_RLNonUni), Grey-Level Nonuniformity at 135 degrees (135dr_GLevNonU), Vertical Grey-Level Nonuniformity (Vertl_GLevNonU), S(2,-2) Entropy, S(3,-3) Entropy, S(5,-5) Entropy, and S(1,-1) Entropy exhibited statistically significant differences (*p* < 0.05) between the group with an unknown cause of abnormal uptake and the group with a known cause of abnormal uptake. Please see Table 7 for a detailed analysis of texture features related to abnormal cardiac uptake in 118 tumor patients using ^18^F-FDG PET/CT imaging.

**Table 7.** **Texture analysis of abnormal cardiac uptake in 118 tumor patients using ^18^F-FDG PET/CT imaging**.

	Unknown cause of abnormal uptake (N = 74)	Known cause of abnormal uptake (N = 44)	*p*-value
S(5,5)Contrast	65.70 ± 53.13	53.69 ± 25.88	0.164
S(0,2)SumAverg	65.89 ± 0.66	65.75 ± 0.54	0.249
Teta3	0.21 ± 0.21	0.31 ± 0.21	0.009
Horzl_RLNonUni	1194.54 ± 675.79	1647.51 ± 729.36	0.001
Teta2	–0.51 ± 0.10	–0.56 ± 0.09	0.016
Horzl_Fraction	0.66 ± 0.08	0.68 ± 0.07	0.404
Teta4	0.38 ± 0.13	0.34 ± 0.12	0.084
S(1,-1)SumEntrp	1.80 ± 0.07	1.82 ± 0.06	0.163
Skewness	–1.06 ± 0.59	–0.99 ± 0.45	0.468
S(3,3)SumVarnc	332.49 ± 58.55	347.69 ± 33.97	0.119
S(1,0)Entropy	2.18 ± 0.13	2.23 ± 0.11	0.034
GrKurtosis	0.35 (–0.40, 2.58)	0.66 (–0.23, 4.03)	0.163
S(5,5)DifVarnc	27.93 ± 21.36	24.30 ± 11.12	0.298
S(5,-5)SumAverg	67.99 ± 1.18	67.87 ± 0.89	0.585
WavEnLH_s-5	465.82 ± 276.78	426.57 ± 245.41	0.439
Kurtosis	1.34 (–0.11, 2.45)	0.93 (0.15, 2.11)	0.270
WavEnLL_s-5	7557.68 ± 4824.90	7220.43 ± 1810.88	0.657
S(0,3)AngScMom	0.0062 ± 0.0056	0.0053 ± 0.0029	0.348
Teta1	0.93 ± 0.06	0.91 ± 0.07	0.071
135dr_RLNonUni	1753.24 ± 966.58	2367.30 ± 1079.81	0.002
45dgr_RLNonUni	1396.44 ± 758.34	1925.52 ± 871.85	0.001
Horzl_GLevNonU	68.49 ± 35.04	95.86 ± 50.90	0.001
45dgr_GLevNonU	76.68 ± 39.99	107.32 ± 59.91	0.001
Vertl_RLNonUni	1250.64 ± 646.87	1664.23 ± 766.32	0.002
135dr_GLevNonU	85.44 ± 44.83	118.36 ± 69.62	0.002
Vertl_GLevNonU	71.33 ± 36.56	97.55 ± 55.30	0.002
S(2,-2)Entropy	2.52 ± 0.13	2.58 ± 0.12	0.012
S(3,-3)Entropy	2.61 ± 0.14	2.67 ± 0.12	0.013
S(5,-5)Entropy	2.63 ± 0.14	2.70 ± 0.13	0.014
S(1,-1)Entropy	2.32 ± 0.13	2.38 ± 0.11	0.015

### 3.8 Multivariate Regression Analysis of Metabolic Parameters and Texture Features for the Cause of Abnormal Cardiac Uptake in Tumor Patients Using ^18^F-FDG PET/CT

A multivariate regression analysis assessed the texture features and metabolic parameters related to abnormal cardiac uptake in 118 tumor patients using ^18^F-FDG PET/CT. The goal was to identify the underlying causes of cardiac uptake irregularities. Included in the analysis were texture features and metabolic parameters that showed significant differences between groups with known and unknown causes. Texture features comprised Teta3, 45dgr_RLNonUni, 45dgr_GLevNonU, and S(1,-1) Entropy, while metabolic parameters encompassed SUVmean-heart and heart-to-blood pool SUV ratio. The findings indicated that Teta3, 45dgr_RLNonUni, 45dgr_GLevNonU, S(1,-1) Entropy, SUVmean-heart, and heart-to-blood pool SUV ratio significantly influenced the specificity of abnormal cardiac uptake lesions. For an in-depth analysis of these features and parameters, please see Table 8 concerning the causes of abnormal cardiac uptake in the aforementioned patient cohort using ^18^F-FDG PET/CT imaging.

**Table 8.** **Multivariate regression analysis of texture features and metabolic parameters for the cause of abnormal cardiac uptake in 118 tumor patients using ^18^F-FDG PET/CT imaging**.

Texture features and metabolic parameters	B	S.E.	*p*	Exp (B)
Teta3	3.398	1.226	0.006	29.907 (2.708, 330.347)
45dgr_RLNonUni	–0.002	0.001	0.036	0.998 (0.995, 1.000)
45dgr_GLevNonU	0.058	0.022	0.008	1.060 (1.015, 1.106)
S(1,-1)Entropy	9.567	2.857	0.001	14,279.265 (52.792, 3,862,281.000)
SUVmean-heart	1.876	0.573	0.001	6.525 (2.123, 20.054)
Heart-to-blood pool SUV ratio	–1.499	0.567	0.008	0.223 (0.073, 0.679)

### 3.9 Discriminative Ability of Texture Features and Metabolic Parameters for Determining the Cause of Abnormal Cardiac Uptake Lesions

The discriminative ability of notable texture features and metabolic parameters, as identified through multivariate analysis, was assessed using ROC curves. The model that integrated Teta3, 45dgr_RLNonUni, 45dgr_GLevNonU, S(1,-1) Entropy, along with SUVmean-heart and heart-to-blood pool SUV ratio, demonstrated superior performance compared to other models relying on these features and parameters. The AUC stood at 0.809 (0.732, 0.886), exhibiting a sensitivity of 77.3% and specificity of 71.6%. For a comprehensive overview of the discriminative performance of these features and parameters in determining the cause of abnormal cardiac uptake lesions, please consult Table 9 and Fig. 3. A bootstrap was used to conduct 1000 times self-sampling for internal validation of the model, yielding a concordance index of 0.808 (0.74, 0.869), as detailed in Fig. 4.

**Table 9.** **Discriminatory efficacy of texture features and metabolic parameters for identifying the cause of abnormal cardiac uptake**.

Texture features and metabolic parameters	AUC	*p*-value	Cut-off value	Sensitivity (%)	Specificity (%)
Teta3	0.678 (0.578, 0.778)	0.001	0.178	86.4	47.3
45dgr_RLNonUni	0.700 (0.603, 0.797)	0.001	1228.877	81.8	55.4
45dgr_GLevNonU	0.672 (0.572, 0.772)	0.002	82.50	61.4	66.2
S(1,-1)Entropy	0.637 (0.536, 0.738)	0.013	2.254	86.4	39.2
SUVmean-heart	0.569 (0.463, 0.675)	0.213	2.550	50	64.9
Heart-to-blood pool SUV ratio	0.467 (0.359, 0.576)	0.552	1.796	70.5	33.8
Combining prediction	0.809 (0.732, 0.886)	0.001	0.339	77.3	71.6

**Fig. 3.**
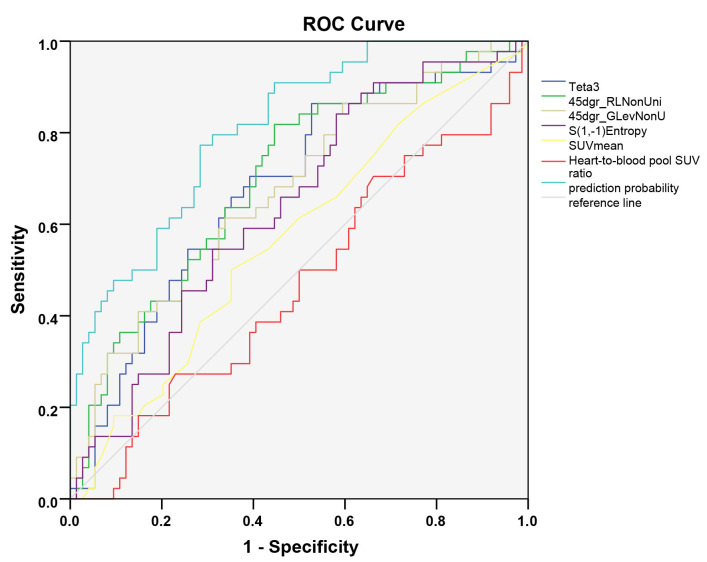
**ROC curve for predicting the cause of abnormal cardiac uptake lesions using texture features and metabolic parameters**.

**Fig. 4.**
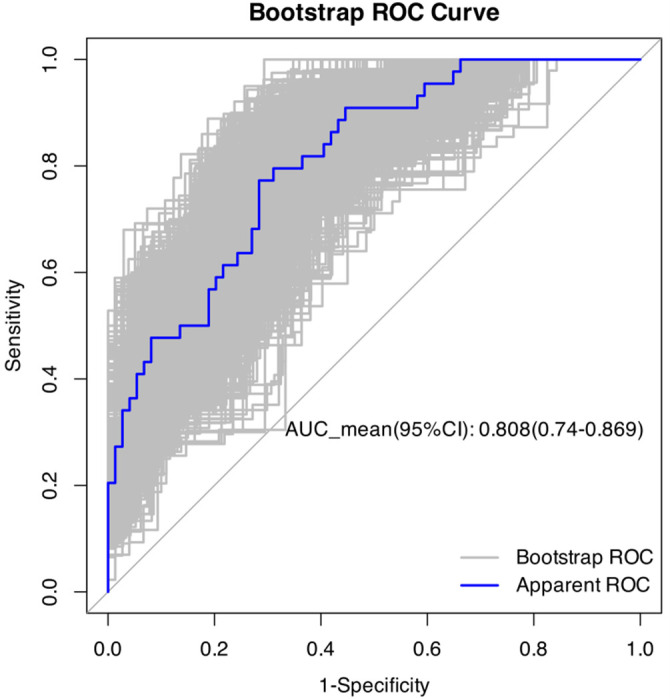
**Internal validation using the bootstrap method of the prediction model**.

## 4. Discussion

Anti-cancer medications can induce cardiotoxicity, manifesting as myocardial inflammation, irreversible damage, and subsequent cardiac impairment [16]. Treatment-related vasoconstriction, along with arterial and venous thrombosis, can precipitate coronary artery disease and acute myocardial infarction [17]. Moreover, treatment effects on blood pressure and the cardiac conduction system may lead to hypertensive crises and severe arrhythmias [18]. Consequently, the European Society of Cardiology guidelines advocate for cardiovascular risk assessment in cancer patients and recommend cardiovascular imaging before and after treatment [19]. Beyond echocardiography, electrocardiography, and cardiac magnetic resonance imaging, nuclear cardiac imaging serves as a prevalent diagnostic tool for assessing drug-induced cardiotoxicity.

While ^18^F-FDG PET/CT imaging primarily targets malignant tumors for staging, assessing burden, and gauging treatment response, its utility has expanded to evaluating cardiotoxic effects of anthracyclines and other anti-cancer agents. These drugs can disrupt mitochondrial oxidative stress metabolism and elevate glycolytic activity in myocardial cells [20]. Given that hexokinase, the rate-limiting enzyme of glycolysis, governs both ^18^F-FDG phosphorylation and retention within cells, heightened myocardial ^18^F-FDG uptake can indicate drug-induced cardiotoxicity [21]. Research by Bauckneht *et al*. [22] demonstrated dose-dependent increases in left ventricular ^18^F-FDG uptake in mice administered varying doxorubicin concentrations. Similarly, a study involving 36 Hodgkin lymphoma patients undergoing ABVD treatment and sequential ^18^F-FDG PET/CT scans revealed elevated left ventricular SUV during doxorubicin therapy, correlating with subsequent cardiac events [22]. Bauckneht *et al*. [22] further noted that lower baseline ^18^F-FDG uptake corresponded with rising doxorubicin dosages, suggesting baseline uptake could influence chemotherapy-linked cardiac toxicity. Additionally, Fathala *et al*. [23] identified a significant association between myocardial ^18^F-FDG uptake patterns—focal or diffuse—and perfusion imaging-based myocardial ischemia, indicating that baseline and treatment-related ^18^F-FDG uptake patterns crucially inform tumor-related cardiac toxicity. However, comprehensive studies analyzing heart uptake patterns before initiating anti-cancer treatment remain limited.

Heart uptake patterns can be categorized as no uptake, diffuse uptake, focal uptake, and focal uptake on a diffuse background. Both no uptake and diffuse uptake are typically deemed physiological. However, specific instances of focal uptake also fall under this category. Notably, uptake patterns remain unaffected by factors like age, blood glucose levels, body weight, or FDG dosage, resulting in poor repeatability in PET/CT examinations [24]. Although focal FDG accumulation in certain regions like the left papillary muscle’s anterior lateral and posterior inferior areas is considered physiological, isolated FDG uptake in this muscle could indicate a tumor or thrombus [25].

The crista terminalis, a smooth muscular band separating the right atrium from its appendage, may occasionally show heightened FDG uptake. This can lead to misinterpretations as myocardial tumors, thrombi, or focal pericardial metastases, despite its physiological nature [26]. Additionally, lipomatous hypertrophy of the interatrial septum (LHIS), a benign condition with a prevalence of around 1–8%, presents challenges due to its appearance as a dumbbell-shaped thickening of the interatrial septum. It displays focal FDG uptake linked to brown adipose tissue, potentially causing confusion with other conditions [27]. Furthermore, basal myocardium regions often manifest circular, semicircular, or spotted FDG uptake, which is another recognized physiological pattern [24].

Given these considerations, this study categorizes cardiac FDG uptake patterns as either physiological or abnormal. Abnormal cardiac uptake may occur in the left ventricle, right ventricle, or atrium. Analyzing patients’ medical histories, cardiac ultrasounds, coronary angiographies, and other imaging studies helped identify prevalent causes of abnormal uptake in various cardiac regions. Specifically, abnormal uptake in the left ventricle commonly stems from myocardial ischemia, hypertensive ventricular hypertrophy, and heart failure. In contrast, abnormal uptake in the right ventricle often relates to conditions like pulmonary arterial hypertension and valvular diseases. Common causes of abnormal uptake in the atrium encompass atrial fibrillation, hypertensive atrial enlargement, and pulmonary arterial hypertension. Elevated pressure or volume loads on the atrium or ventricle trigger the synthesis and upregulation of glucose transporters, culminating in heightened myocardial cell metabolism [28]. In scenarios involving ventricular hypertrophy, augmented myocardial workload can further amplify myocardial cell metabolism. Additionally, increased FDG uptake observed in atrial fibrillation patients may correlate with enhanced epicardial adipose tissue activity [29].

Upon analyzing abnormal cardiac uptake causes among tumor and non-tumor patients, it became evident that tumor patients exhibit a significantly higher rate of unexplained cardiac uptake compared to their non-tumor counterparts. Given this heightened abnormal cardiac uptake in tumor patients, a plausible link to tumor burden emerges. Further stratifying tumor patients by tumor burden using ^18^F-FDG PET/CT revealed that the high TLG group manifested a notably elevated rate of unexplained cardiac uptake relative to the low TLG group. Consequently, unexplained cardiac uptake in tumor patients undergoing ^18^F-FDG PET/CT likely correlates with tumor burden, suggesting that increased tumor burden elevates the incidence of abnormal cardiac uptake [30]. Unexplained cardiac uptake may be the manifestation of tumor-associated myocardial metabolic remodeling, which essentially reflects a dual process of adaptive compensation and early injury of the myocardium under the stimulation of the tumor microenvironment or damage [31]. Tumors sequester peripheral glucose through the Warburg effect while inhibiting myocardial fatty acid oxidation. To compensate for the energy deficit, the myocardium upregulates glucose transporter 4 (GLUT4) expression and activates the PI3K/Akt pathway to enhance glucose uptake and glycolysis. In this context, increased glucose uptake represents a compensatory adaptation of the myocardium to energy substrate imbalance. Reactive oxygen species (ROS) released by tumors cause mild damage to myocardial mitochondria, leading to reduced oxidative phosphorylation efficiency [32]. Consequently, the myocardium actively switches to glycolysis for energy production—even under oxygen-sufficient conditions. Here, increased glucose uptake serves as a metabolic “risk-aversion” strategy after mitochondrial dysfunction, preventing energy crisis triggered by reliance on the defective oxidative phosphorylation pathway. While these findings align with prior research, the precise underlying mechanism remains elusive.

In-depth analysis comparing unknown cause abnormal cardiac uptake to known cause abnormal cardiac uptake in tumor patients revealed no notable differences in metabolic parameters such as SUVmax and SUVmean between the groups. SUV merely indicates individual pixel metabolic levels within images, lacking the capacity to encapsulate the intricate details or overall status of abnormal cardiac uptake lesions. Thus, solely gauging changes in SUV values falls short in distinguishing between known and unknown causes of abnormal cardiac uptake.

Conversely, texture analysis unveils nuanced image information imperceptible to the unaided eye by delineating relationships among voxels and pixels concerning grayscale, intensity, and spatial positioning. This analytical approach equips clinicians with the tools to discern subtle disparities among akin lesions. Despite limited research on texture analysis of abnormal cardiac uptake lesions, this study determined that integrating Teta3, 45dgr_RLNonUni, 45dgr_GLevNonU, S(1,-1)Entropy, SUVmean, and heart-to-blood pool SUV ratio within a predictive model yielded optimal discriminatory outcomes, boasting an AUC of 0.809 alongside elevated sensitivity and specificity. This underscores the efficacy of combining texture features with metabolic parameters in ^18^F-FDG PET/CT for pinpointing abnormal cardiac uptake causes.

Notably, uniformity metrics from the run length matrix and gray level matrix in texture analysis can signify lesion pixel consistency, with the known cause abnormal uptake group exhibiting markedly greater non-uniformity than its unknown cause counterpart. Elevated values indicate increased image irregularity. Furthermore, when assessing lesion metabolic parameters, a heightened ratio of lesion SUVmax to corresponding blood pool SUVmax augments the probability of identifying known cause abnormal uptake. This comprehensive approach emphasizes that amalgamating texture with metabolic parameters enhances predictive accuracy for abnormal cardiac uptake causes. Crucially, this method may provide some assistance in early identification of the underlying causes of abnormal cardiac uptake in patients during follow-up ^18^F-FDG PET/CT, before other imaging evidence of organic damage emerges.

## 5. Limitations

This study presents several limitations worth noting. Firstly, being a retrospective clinical study, it lacks the capability to delve into the pathological or histological dimensions of abnormal cardiac uptake of ^18^F-FDG in patients, thereby hindering a clear understanding of its underlying mechanisms. Secondly, despite incorporating an extended fasting period (≥12 hours) in the imaging protocol, complete suppression of physiological uptake of ^18^F-FDG by the left ventricular myocardium may not have been achieved. Thirdly, simultaneous myocardial fatty acid imaging was absent from the study.

While ^18^F-FDG PET/CT imaging remains pivotal for diagnosing, staging, and assessing treatment responses in tumors, its role extends to evaluating cardiac abnormalities manifesting through abnormal ^18^F-FDG uptake. Although both unknown cause and known cause abnormal cardiac uptakes may correlate with tumor burden, the latter is more likely associated with conditions like myocardial ischemia, hypertension, and atrial fibrillation. Distinguishing between these two necessitates more than just metabolic parameters; it demands a comprehensive assessment integrating both metabolic parameters and texture features. Such an approach furnishes a theoretical framework for elucidating the emergence of new cardiac abnormal uptakes and alterations in cardiac abnormal uptake patterns during tumor treatments.

## 6. Conclusion

This study analyzed the possible causes and characteristics of myocardial abnormal uptake in tumor patients during their initial ^18^F-FDG PET/CT imaging, providing theoretical support for understanding changes in cardiac abnormal uptake during tumor treatment. It is expected to offer additional value in observing tumor-related cardiac toxicity through ^18^F-FDG PET/CT imaging.

## Availability of Data and Materials

The datasets generated and analysed during the current study are available from the corresponding author upon reasonable request.
